# P04.58. Inner congruence, mindfulness and positive mood in experienced yoga practitioners

**DOI:** 10.1186/1472-6882-12-S1-P328

**Published:** 2012-06-12

**Authors:** A Hedtstück, T Ostermann, A Büssing

**Affiliations:** 1 University of Witten/Herdecke, Herdecke, Germany

## Purpose

Besides physical movements, yoga involves mental techniques which are considered to be essential for its effectiveness. However, active participation of the practitioners seems to be essential. This attitude can be operationalized and measured with the “Inner Congruence and Peaceful Harmony” (ICPH) questionnaire. In a previous study, we have shown that ICPH can be predicted solely by the acceptance component of mindfulnes. Now we investigated whether and how variables of mental stability change with respect to ICPH.

## Methods

Prospective analysis of data from 160 individuals participating in a two-year yoga teacher training (Yoga Vidya: 91% women; mean age 41±8 years; mean duration of yoga practice 39±53 months). Standardized questionnaires were administered at the start (t1), 3 months (t2), and 6 months (t3) later which comprised of Inner Congruence and Peaceful Harmony (ICPH), Mindfulness (FMI), Life Satisfaction (BMLSS), Positive Mood States (POMS/ASTS), health related Quality of Life (SF-12), Light-Heartedness / Easiness (LHE), and Aspects of Spirituality (ASP).

## Results

During the course of intensified yoga practice, particularly LHE (Cohen´s d=.73) and mindfulness (d=.58) increased significantly (p<.0001; Friedman), while positive mood (d=.29), mental health (d=.27) and ICPH (d=.22) increased only slightly. With respect to ASP, conscious interactions (ASP; d=.34) and religious orientation (ASP, d=.34) increased significantly (p<.01). Individuals with primarily low ICPH scores (28%) showed a significant development in mindfulness (d=.78) and LHE (d=.77), while those with moderate ICPH (53%) had a small increase of mindfulness (d=.47), but a strong increase in LHE (d=.93). Those with primarily high ICPH (19%) showed only small increases in mindfulness (d=.35) and LHE (d=.42).

## Conclusion

Even in already experienced yoga practitioners, positive mood and mindfulness increased significantly. One could suggest that ICPH represents a trait which may be developed to facilitate the beneficial effects of mindfulness on mental stability. Further investigations enrolling patients with chronic diseases are required.

